# Mammalian tolloid proteinases: role in growth factor signalling

**DOI:** 10.1002/1873-3468.12287

**Published:** 2016-07-22

**Authors:** Helen Troilo, Christopher P. Bayley, Anne L. Barrett, Michael P. Lockhart‐Cairns, Thomas A. Jowitt, Clair Baldock

**Affiliations:** ^1^ Wellcome Trust Centre for Cell‐Matrix ResearchFaculty of Life SciencesUniversity of ManchesterUK; ^2^ Beamline B21Diamond Light SourceHarwell Science & Innovation CampusDidcotOxfordshireUK

**Keywords:** BMP signalling, chordin, latent TGFβ‐binding protein, twisted gastrulation

## Abstract

Tolloid proteinases are essential for tissue patterning and extracellular matrix assembly. The members of the family differ in their substrate specificity and activity, despite sharing similar domain organization. The mechanisms underlying substrate specificity and activity are complex, with variation between family members, and depend on both multimerization and substrate interaction. In addition, enhancers, such as Twisted gastrulation (Tsg), promote cleavage of tolloid substrate, chordin, to regulate growth factor signalling. Although Tsg and mammalian tolloid (mTLD) are involved in chordin cleavage, no interaction has been detected between them, suggesting Tsg induces a change in chordin to increase susceptibility to cleavage. All members of the tolloid family bind the N terminus of latent TGFβ‐binding protein‐1, providing support for their role in TGFβ signalling.

## Abbreviations

**BMP‐1**, bone morphogenetic protein‐1

**CR**, cysteine‐rich

**ECM**, extracellular matrix

**EGF**, epidermal growth factor

**LAP**, latency‐associated protein

**LLC**, large latent complexes

**MMPs**, matrix metalloproteinases

**mTLD**, mammalian tolloid

**ONT1**, olfactomedin 1

**PCPE‐1**, procollagen C‐endopeptidase enhancer‐1

**sFRP2**, secreted frizzled‐related protein 2

**SLC**, small latent complex

**TGF**, transforming growth factor

**TLL**, tolloid‐like

**Tsg**, twisted gastrulation

**vWFC**, von Willebrand Factor type C

**Figure 1:**
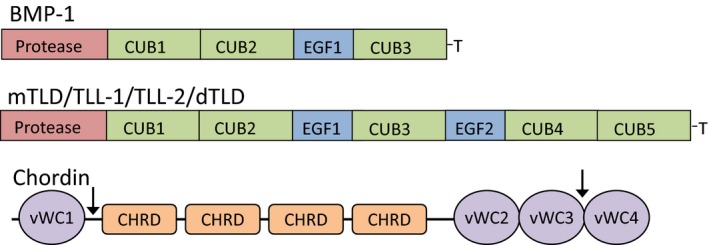
Schematic diagram of the domain structures of tolloid family members and chordin. The tolloids are composed of a protease domain followed by CUB and EGF domains. BMP‐1 lacks the last three noncatalytic EGF and CUB domains. ‘T’ represents a unique C‐terminal sequence. Chordin is composed of four von Willebrand factor type C domains and chordin specific or CHRD domains. Tolloids cleave chordin after vWC domains ‐1 and ‐3 (indicated by an arrow).

The mammalian tolloid family consists of four members: bone morphogenetic protein‐1 (BMP‐1), mammalian tolloid (mTLD) which are alternatively spliced products of the Bmp1 gene 1 and two genetically distinct proteins tolloid‐like (TLL)‐1 and TLL‐2 2, 3. Together they comprise a small group of zinc and calcium dependent proteinases 4. BMP‐1 is not a member of the BMP family of cytokines, it was initially copurified with BMP2 and BMP3 from extracts of bone and named accordingly 5, however, it had also been previously identified as procollagen C‐proteinase 6. The domain organization of mTLD, TLL‐1 and TLL‐2 is identical and this is evolutionarily conserved, for example, in Drosophila (dTLD) and Xenopus (Xld) 7. This arrangement consists of an N‐terminal protease domain, making tolloids part of the astacin superfamily 7, followed by up to five CUB (Complement, Uegf and BMP‐1) modules and two calcium ion binding epidermal growth factor (EGF)‐like domains (Fig. 1).

Interestingly, BMP‐1 (the shorter splice variant), which is generally the most active tolloid and cleaves a wide range of substrates, is expressed without the final three noncatalytic domains. It was shown through biophysical and structural methods that mTLD and TLL‐1 form noncovalently linked Ca^2+^‐dependent dimers in solution, whereas BMP‐1 and TLL‐2 remain as monomers 8, 9, 10. It has been demonstrated that substrate exclusion due to dimerization results in reduced activity of these proteinases in comparison to BMP‐1 9, 10. However, TLL‐2 is predominantly monomeric in solution so its activity must be modulated by another mechanism. The noncatalytic domains appear to function by restricting proteolytic activity both in terms of substrate specificity 11 and efficiency by exosite binding 9. Additionally, C‐terminal truncation of TLL‐2 and *drosophila* TLD (dTLD) results in the loss of activity 8, 11, 12. However, C‐terminal truncation of TLL‐1 and mTLD increases their activity against some substrates 9, 10, 13. Furthermore, when secreted alone *in vitro*, the BMP‐1/mTLD protease domain cleaves additional sites in previously characterized substrates and also cleaves other matrix proteins such as fibronectin, which are left intact by the full‐length protease 13. BMP‐1/mTLD act on a wide‐range of substrates including extracellular matrix precursors and BMP/TGFβ regulators.

### Tolloid substrates

**Figure 2:**
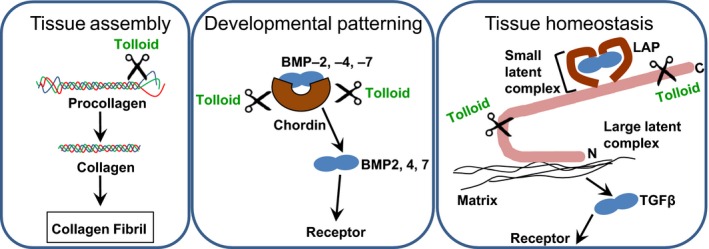
Overview of the key roles tolloid proteinases play in processing ECM molecules such as cleaving the C‐propeptide from procollagen in normal tissue assembly; in regulating growth factor signalling by cleaving BMP antagonist chordin during developmental patterning and latent TGFβ‐binding protein to maintain tissue homoeostasis.

In vertebrates, BMP‐1/mTLD proteinases are involved in the biosynthetic processing of a diverse range of extracellular matrix (ECM) precursors required for laying down the extracellular matrix and normal tissue assembly (Fig. 2). Substrates include the major and minor fibrillar procollagens 14, 15, 16, the collagen and elastin crosslinking enzyme prolysyl oxidase 17, cellular anchoring proteins prolaminin‐5 and procollagen VII 18, 19 and the small leucine‐rich proteoglycans, osteoglycin and probiglycan 20, 21. Mutations in BMP‐1/mTLD have been shown to cause osteogenesis imperfecta (OI), a disease primarily characterized by fragile bones that have a high susceptibility to fracture, along with neurological impairments 22, 23. Martinez‐Glez *et al*. reported that the F249L missense mutation in BMP‐1/mTLD decreased the ability for procollagen I C‐propeptide to be processed correctly resulting in the OI phenotype 23 demonstrating the essential role of tolloids in collagen processing. In addition to processing precursor proteins to their mature form, cleavage of mature proteins by tolloids can give rise to fragments with novel biological functions, for example, cleavage of endorepellin gives rise to the angiostatic LG3 fragment 24. BMP‐1/mTLD proteinases are also instrumental in the release a number of transforming growth factor (TGF)‐β superfamily members from inhibitory complexes, including BMP‐2, ‐4 and 7, growth and differentiation factors 8/11 and TGFβ1. This action modulates developmental patterning, growth of skeletal muscle, and tissue homoeostasis respectively 3, 25, 26, 27.

### Role of tolloids in BMP and TGFβ signalling

Through the proteolytic cleavage of their substrates BMP‐1/mTLD modify matrix components thereby regulating many cellular activities such as proliferation and differentiation. BMP‐1/mTLD are involved in the regulation of dorso‐ventral patterning through the cleavage of the BMP antagonist chordin (Fig. 2). BMPs are a group of pivotal morphogenetic signals, orchestrating tissue architecture throughout the body. They were not only identified for their function in bone and cartilage formation but also have roles in patterning, kidney, eye and heart formation 28, 29. Chordin functions as an extracellular antagonist of BMP‐2, ‐4 and ‐7 by binding to them and preventing their interaction with the BMP receptors 30. Cleavage of the BMP‐chordin complex by BMP‐1/mTLD proteinases liberates BMP, resulting in downstream signalling events including the generation of BMP gradients. In addition, tolloids are involved in the proteolytic release mechanism of active TGFβ1 growth factor from the latent complexes by providing the precipitating cleavage events which leave the remaining complex susceptible to MMPs 25, 31.

### Tolloid enhancers

The activity of BMP‐1/mTLD is modified by substrate‐specific enhancers. Procollagen C‐endopeptidase enhancer‐1 (PCPE‐1) is a CUB domain containing protein that enhances cleavage of fibrillar procollagens by BMP‐1/TLD, however, PCPE‐1 does not enhance cleavage of other tolloid substrates 32. PCPE‐1 binds directly to procollagen and cleavage of procollagen by mammalian tolloid is enhanced by as much 10‐fold by PCPE‐1 33. To add a further layer of complexity, PCPE binds heparan sulphate proteoglycans (such as syndecans) 34 and BMP1 procollagen cleavage can be super‐stimulated by heparan sulphate 35. These data suggest that scaffolding at the cell surface by heparan sulphate proteoglycans might allow accelerated procollagen processing by BMP1/mTLD and thus collagen assembly. Twisted gastrulation (Tsg) is a noncatalytic ECM glycoprotein important for skeletogenesis 36 and maintaining bone mineral density 37. Tsg can act as a BMP antagonist by enhancing chordin/BMP complex formation 38, 39, 40. Interestingly, Tsg can also act as a BMP agonist, by enhancing the cleavage of chordin relieving the inhibition of BMP signalling 41. Other examples include the scaffolding protein, Olfactomedin 1 (ONT1), which functions by binding to both tolloid and substrate, bringing them into close proximity to enhance the rate of cleavage 42 and secreted frizzled‐related protein 2 (sFRP2) which enhances procollagen proteinase activity in mammals 43. However, in xenopus and zebrafish the sFRP, Sizzled, was shown to inhibit tolloid processing of chordin 44, 45, indicating that the function of these regulators is not conserved.

This review will discuss the role of tolloid family proteases in the regulation of TGFβ‐superfamily growth factor signalling. Specifically (1): how tolloid cleavage of chordin is enhanced by twisted gastrulation to result in the release of active growth factor, and (2) the role of tolloid cleavage of the large latent TGFβ complex in proteolytic activation of TGFβ1. Through these pathways tolloids function not only by activating matrix proteins following secretion but also as vital regulators in development and homoeostasis.

## Enhancing tolloid cleavage by twisted gastrulation

### Role of tolloid as a BMP agonist

The TGFβ/BMP signalling pathways control a myriad of events, including cell proliferation, differentiation, apoptosis, migration, ECM remodelling and tumour invasion/metastasis 46. During embryogenesis of vertebrates and invertebrates, antagonism between BMPs and chordin is a general mechanism by which the dorso‐ventral axis is established 47. Chordin, BMPs and Tsg form a tripartite complex which can diffuse through the extracellular space. While BMPs are bound to chordin they are unable to bind to their cell surface receptors (BMPR) type I and II 30, 48, 49. Cleavage of chordin by tolloids allows for BMPs to be released exerting a dorsal‐ventral patterning effect, in vertebrates 3, 50 and other organisms 51, 52, 53 through the SMAD or MAD pathways. In addition to its important developmental role, chordin is also involved in adult processes as it is expressed by chondrocytes during cartilage formation following bone fracture 54. Chordin also has a role in the osteoarthritic process as higher protein levels are found in osteoarthritis than in normal cartilage 55.

### Cleavage of chordin by tolloid family proteinases

Chordin, a 100 kDa glycoprotein, has a modular domain architecture consisting of four domains homologous to von Willebrand Factor type C (vWFC) domains, sometimes also referred to as Cysteine‐Rich (CR) domains, and four chordin specific or CHRD domains which are also cysteine‐rich (Fig. 1). Chordin adopts a horseshoe shaped structure in which the four CHRD domains separate the first and second vWFC domains 56. The first and third vWFC domains bind to BMP‐2 and ‐4 48, and the first and fourth vWFC domains bind BMP‐7 49. The CHRD domains act as spacers, supporting a horseshoe shaped structure of chordin which facilitates simultaneous binding by the N‐ and C‐terminal vWFC domains to the BMP ligand 56. Cleavage of chordin by tolloid proteinases occurs at two specific interdomain sites following the first and third vWFC domains 57. BMP‐1 and TLL‐1 cleave chordin with the greatest efficiency 3, 9, 10, whereas mTLD and TLL‐2 are less active 8. However, the noncatalytic domains of all mammalian tolloids bind to chordin with high affinity 8.

Since the biological activity of the individual vWFC domains is 5‐ to 10‐fold lower than full‐length chordin, it has been speculated that tolloid cleavage would release lower affinity vWFC‐BMP complexes 48. However, the affinity of the cleavage fragments is very similar to full‐length chordin, and these fragments retain or even enhance BMP inhibitory activity, suggesting cleavage of both sites may be required for ablation of BMP inhibition by chordin 56, 58, 59, 60. One role for chordin is that beyond simply sequestering BMP in the tissue where it is expressed, chordin facilitates diffusion of BMP to other tissues 61. The result is localized build‐up of inactive BMP, in preparation for tolloid cleavage. This allows spatially and temporally controlled liberation of BMP from this complex allowing localized pockets of BMP activity at the dorsal pole of the embryo 62, 63. This model is supported by research in *Drosophila*, where a complex of the chordin‐BMP‐TLD homologues is assembled on collagen IV and mobilized by Tsg 11, 64.

### Enhancement of tolloid cleavage by Tsg

Tsg is a 33 kDa monomeric glycoprotein 59, identified as essential for the correct formation of the dorsal‐ventral axis 65. It is important for skeletogenesis 36 and maintaining bone mineral density in adulthood 37. It has two cysteine‐rich domains, one of which is homologous to vWFC domains 66. Tsg has been shown to act in both a pro‐BMP and anti‐BMP manner. Tsg can act as a BMP‐antagonist by binding to both chordin and BMP, enhancing chordin‐BMP complex formation 38, 39, 40. Consistent with this function, Tsg potentiates chordin's ability to induce a secondary axis in Xenopus embryos 38. Tsg binds to the chordin vWFC‐2 and ‐3 domains with high‐affinity and interacts more weakly to vWFC‐1 and ‐4 59.

Tsg acts as a BMP agonist by enhancing the cleavage of chordin by tolloid proteinases 41, 67. Tsg does not bind directly to tolloid proteinases and *in vitro* it can enhance tolloid cleavage of chordin in the absence of other factors so it must potentiate this enhancing effect through interaction with chordin 59. There is evidence that Tsg may induce conformational changes in chordin that lead to increased cleavage. Mouse chordin has a third tolloid cleavage site in addition to the two highly conserved sites. Cleavage of this third site was only observed *in vitro* in the presence of Tsg 38, suggesting that this is a cryptic cleavage site inaccessible to tolloid proteinases in the absence of Tsg. Similarly, the presence of Tsg also alters the cleavage fragments observed following cleavage of the *Drosophila* chordin homologue Sog by dTLD *in vitro* 68. The tolloid proteinases appear key to this switch in BMP regulation by Tsg, as was supported by RNA injection experiment studies in *Xenopus*. In dorsalized *Xenopus* embryos, the injection of Xolloid or Tsg mRNA rescues the formation of ventral trunk‐tail structures normally seen in regions of high BMP signalling 41. However, on coinjection with dominant negative Xolloid mRNA, Tsg mRNA loses its pro‐BMP ventralizing ability 41.

In mammals, Tsg is strongly expressed in cartilage and is involved in chondrocyte differentiation, playing an important role in cartilage development 69. Tsg null mice display a dwarfism phenotype and osteopenia, due to defective chondrogenesis and endochondral ossification 36, 70, 71. The tolloid metalloproteinases are known to be a key to bone and cartilage formation, and BMP‐1, mTLD and TLL‐1 are expressed in developing bone and cartilage in mice 3. In the chick upregulation of *tolloid* gene expression precedes chrondogenic differentiation 72, 73. Indeed, BMP‐1 can induce ectopic cartilage formation *in vivo* 5. Hence, Tsg appears to be important during cartilage formation due to its promotion of BMP signalling via the enhancement of tolloid metalloproteinase activity. The importance of Tsg as a tolloid enhancer is highlighted by the pathologies that result from its absence.

## Tolloid cleavage of the large latent TGFβ complex

### Role of tolloids in TGFβ signalling

All TGFβ isoforms can be secreted as large latent complexes (LLC) which are not able to activate downstream signalling. They consist of three components: a disulphide bonded homodimer of mature TGFβ, associated noncovalently with its latency‐associated protein (LAP) which together comprise the small latent complex (SLC). The SLC is covalently linked by a disulphide bond to latent TGFβ‐binding protein (LTBP) 74, 75. LTBPs are large extracellular matrix modular glycoproteins 76 with an important role in the processing and secretion of TGFβ. In many cell types, the expression of LTBP1 is coregulated with TGFβ1 77 and a lack of LTBP‐1 or ‐3 directly correlates with decreased TGFβ activation 78, 79. In addition, LTBP1 targets TGFβ to the ECM and is covalently linked to extracellular matrix fibrils by transglutaminase‐2 cross‐links 80.

Interestingly, the methods through which tolloids activate TGFβ differ from the mechanisms through which they regulate BMPs. Unlike chordin, LAP is not itself a tolloid substrate, nevertheless tolloids have a key role in regulating TGFβ activation, contributing to the release of latent TGFβ from the extracellular matrix through cleavage of LTBP‐1 25. It also deactivates the soluble form of the TGFβ coreceptor betaglycan through proteolytic cleavage, thereby increasing TGFβ bioavailability 81. Active TGFβ is a potent inducer of tolloid expression and it is expected that this contributes to a positive feedback loop of TGFβ signalling in inflammation and fibrosis 25.

### Activation of TGFβ by proteases and integrins

A variety of physiological methods of releasing TGFβ from the LLC have been suggested (for review see 82). Integrin‐mediated activation appears to have a major role. LAPs from TGFβ1 and ‐3 have integrin‐binding motifs and mutation of this sequence in TGFβ1 phenocopies the TGFβ1 null mice 83. Integrin αvβ6 binds and activates TGFβ but interaction of the LLC with fibronectin is required 84. Force unfolding of LAP is thought to be the underlying mechanism in integrin activation events 85, 86. A short region in the N terminus of LTBP1 (amino acid residues 402–529) coupled to the C‐terminal TGFβ1‐binding domain of LTBP1 is sufficient to permit activation 87 suggesting that simultaneous binding between integrins to LAP and LTBP1 to other matrix components is required.

Several proteases have also been implicated in activating latent TGFβ, including matrix metalloproteinases (MMPs) 88 and tolloid proteinases 25. The recently solved structure of SLC shows that protease sensitive sites on LAP are surface accessible 85. Tolloids cleave the LLC at two sites on LTBP1 but do not cleave LAP. Following tolloid cleavage, LAP (still bound to the LLC) is a substrate for MMPs, the action of which may subsequently release TGFβ 25, 31. Interestingly, in the absence of TGFβ, BMP‐1 did not cleave LTBP‐1 but only when it was part of the LLC.

### Mammalian tolloids bind to the N‐terminal region of LTBP1

**Figure 3:**
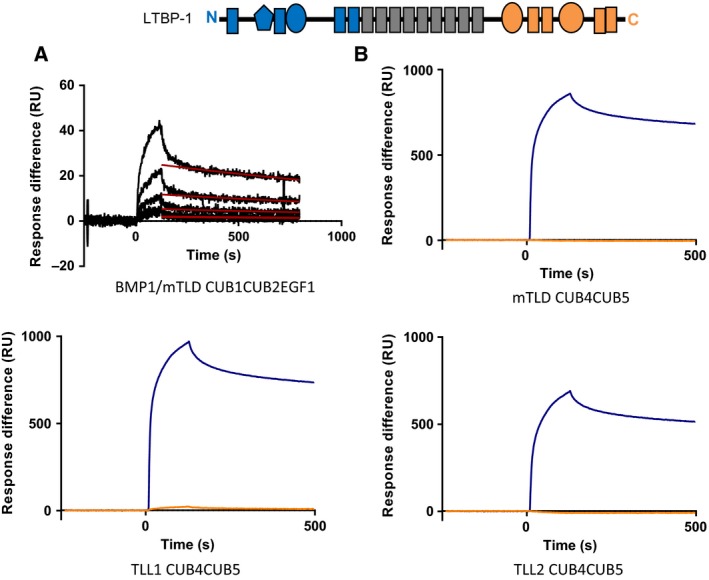
Surface Plasmon resonance binding analysis of interactions between LTBP1 and mammalian tolloids. Domain structure of human LTBP1. EGF domains are shown as rectangles, TGFβ binding‐like (TB) domains as ovals and a hybrid EGF/TB domain is represented as a pentamer. The LTBP1 N‐terminal (NT) and C‐terminal (CT) constructs are shown coloured in blue and orange respectively. (A) Binding analysis of full‐length LTBP1 to immobilized protein fragment CUB1CUB2EGF1 from BMP‐1/mTLD. Analyte concentrations = 0–40 nm. The real‐time binding curves are shown in black and the model of Langmuir off‐rate analysis is shown in red. Experiments performed in triplicate, representative curves shown. (B) LTBP1 NT and LTBP1 CT regions binding to immobilized CUB4CUB5 from mTLD, TLL‐1 or TLL‐2. LTBP1 NT binding in blue, CT binding in orange. Analyte concentration = 500 nm. Methods and experimental details are reported in 10.

Since LTBP1 has previously been identified as a tolloid substrate, binding of full‐length LTBP1 to the noncatalytic domains (CUB1CUB2EGF1) of BMP‐1/mTLD was analysed by surface plasmon resonance. These data showed that BMP‐1/mTLD bound to LTBP‐1 (Fig. 3). To determine whether both N‐ and C‐terminal regions of LTBP1 interacted with tolloids, these regions of LTBP‐1 were screened for binding to the CUB4CUB5 domains of TLL‐2, TLL‐1 and mTLD. For the C‐terminal LTBP‐1 region low or no binding was detected to any proteinase. However, the N‐terminal region of LTBP1 showed a stronger response (Fig. 3). This suggests that the C‐terminal cleavage site either binds to other tolloid domains, or that tolloid binds exclusively at the N terminus and flexibility in LTBP‐1 allows the protease access to both cleavage sites.

Consistent with previous findings BMP‐1, mTLD and TLL‐2 were unable to cleave LTBP1 when not covalently associated with LAP as part of the large latent complex (not shown). LTBP‐1 is frequently expressed in the absence of the SLC and some members of the LTBP family are unable to bind SLC 89. As cleavage by tolloids is specific to the LLC rather than free LTBP suggests that its regulatory role is targeted to the TGFβ pathway rather than also regulating TGFβ‐independent functions of LTBPs 90, 91, 92, 93.

## Conclusions and perspectives

Tolloid family metalloproteases are key activators of TGFβ family of signalling molecules, an effect which is exerted through direct cleavage of inhibitors such as chordin and indirectly through cleavage of LTBPs. This role is regulated by modulators like Tsg resulting in precision in the activation of these signalling pathways. The tolloid family exert such a broad influence over matrix deposition and homeostasis that this is a promising pathway for future therapeutic intervention, for example, in cancers and bone disorders, however, it needs to be better understood. Further structural study of this family, in particular in complex with its binding partners is needed to enhance our knowledge of its regulation and context‐dependent specificity.

## Author contributions

HT, ALB, MPLC and CB wrote the paper. CPB and TAJ analysed data shown in Figure 3.
